# Methyl 4-anilino-2′,5-dioxo-1′,2′-di­hydro-5*H*-spiro­[furan-2,3′-indole]-3-carboxyl­ate

**DOI:** 10.1107/S1600536813014967

**Published:** 2013-06-08

**Authors:** Rajeswari Gangadharan, K. Sethusankar, Selvarangam E. Kiruthika, P. T. Perumal

**Affiliations:** a Department of Physics, Ethiraj College for Women (Autonomous), Chennai 600 008, India; b Department of Physics, RKM Vivekananda College (Autonomous), Chennai 600 004, India; c Organic Chemistry Division, Central Leather Research Institute, Adyar, Chennai 600 020, India

## Abstract

In the title compound, C_19_H_14_N_2_O_5_, the spiro junction links an oxindole moeity and a furan ring, which subtend a dihedral angle of 83.49 (6)°. The mol­ecular structure features an N—H⋯O hydrogen bond, which generates an *S*(6) ring motif. The crystal packing is governed by two N—H⋯O inter­actions, one of which generates a centrosymmetric *R*_2_^2^(14) dimer. The other N—H⋯O inter­action along with a C—H⋯O hydrogen bond contributes to the formation of a *C*_2_^2^[*R*_2_^2^(9)] dimeric chain running along the *b-*axis direction.

## Related literature

For applications of spiro oxindoles, see: Kornet & Thio (1976 ▶); Kobayashi *et al.* (1991 ▶). For applications of furans, see: Schoop *et al.* (2000 ▶). For puckering and asymmetry parameters, see: Cremer & Pople (1975 ▶). For a related structure, see: Gayathri *et al.* (2006 ▶). For graph-set notation, see: Bernstein *et al.* (1995 ▶).
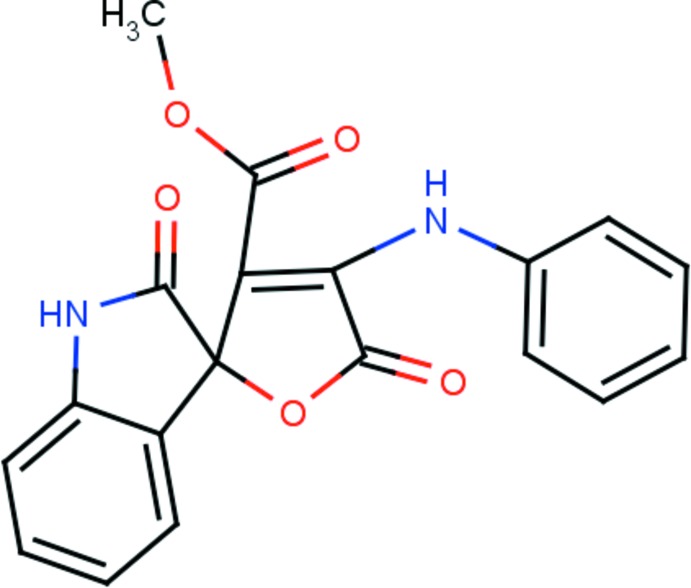


## Experimental

### 

#### Crystal data

C_19_H_14_N_2_O_5_
*M* *_r_* = 350.32
Monoclinic, 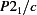

*a* = 12.1713 (6) Å
*b* = 13.6144 (7) Å
*c* = 10.9602 (6) Å
β = 114.813 (2)°
*V* = 1648.50 (15) Å^3^
*Z* = 4
Mo *K*α radiation
μ = 0.10 mm^−1^
*T* = 296 K
0.30 × 0.25 × 0.20 mm

#### Data collection

Bruker SMART APEXII CCD diffractometer
Absorption correction: multi-scan (*SADABS*; Bruker, 2008 ▶) *T*_min_ = 0.969, *T*_max_ = 0.979
20901 measured reflections
5167 independent reflections
3502 reflections with *I* > 2σ(*I*)
*R*_int_ = 0.026

#### Refinement

*R*[*F* ^2^ > 2σ(*F* ^2^)] = 0.044
*wR*(*F* ^2^) = 0.132
*S* = 1.00
5167 reflections
242 parameters
2 restraints
H atoms treated by a mixture of independent and constrained refinement
Δρ_max_ = 0.26 e Å^−3^
Δρ_min_ = −0.16 e Å^−3^

### 

Data collection: *APEX2* (Bruker, 2008 ▶); cell refinement: *SAINT* (Bruker, 2008 ▶); data reduction: *SAINT*; program(s) used to solve structure: *SHELXS97* (Sheldrick, 2008 ▶); program(s) used to refine structure: *SHELXL97* (Sheldrick, 2008 ▶); molecular graphics: *ORTEP-3 for Windows* (Farrugia, 1997 ▶); software used to prepare material for publication: *SHELXL97* and *PLATON* (Spek, 2009 ▶).

## Supplementary Material

Crystal structure: contains datablock(s) global, I. DOI: 10.1107/S1600536813014967/rk2403sup1.cif

Structure factors: contains datablock(s) I. DOI: 10.1107/S1600536813014967/rk2403Isup2.hkl

Supplementary material file. DOI: 10.1107/S1600536813014967/rk2403Isup3.cml

Additional supplementary materials:  crystallographic information; 3D view; checkCIF report

## Figures and Tables

**Table 1:** Hydrogen-bond geometry (Å, °)

*D*—H⋯*A*	*D*—H	H⋯*A*	*D*⋯*A*	*D*—H⋯*A*
N1—H1*A*⋯O5^i^	0.89 (2)	2.19 (2)	3.0268 (16)	157 (2)
N2—H2*A*⋯O1^ii^	0.89 (1)	2.19 (1)	2.9907 (17)	149 (1)
N2—H2*A*⋯O5	0.89 (1)	2.39 (2)	2.9691 (16)	123 (1)
C13—H13*A*⋯O1^iii^	0.96	2.41	3.2999 (18)	153

Symmetry codes: (i) 
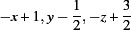
; (ii) 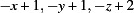; (iii) 
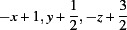
.

**Fig. 1.**
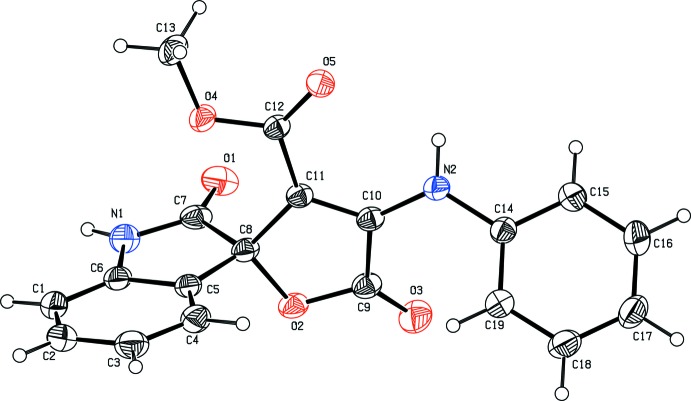
The molecular structure of the title compound with the atom numbering scheme. Displacement ellipsoids are drawn at 30% probability level. H atoms are present as small spheres of arbitary radius.

**Fig. 2.**
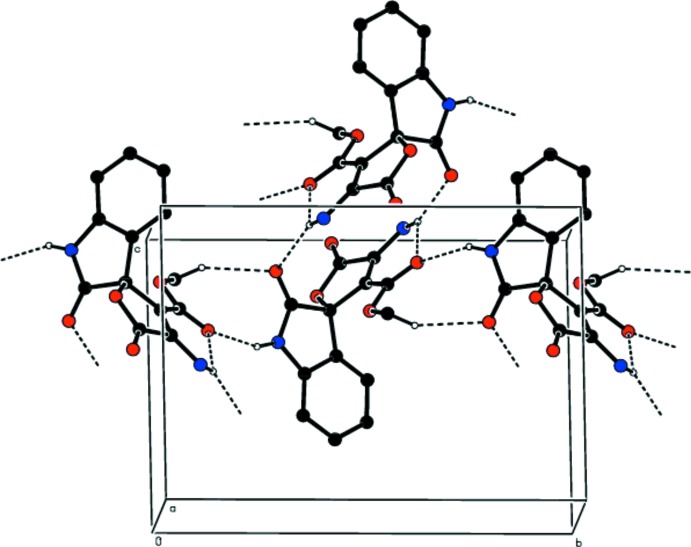
Part of crystal structure of the title compound, showing the formation the intramolecular S(6) ring motif, R22(14) centrosymmetric and the R22(9) non–centrosymmetric dimers as viewed along the b–axis.

## supplementary crystallographic information

### Comment

Spiro compounds represent an important class of naturally occurring substances characterized by highly pronounced biological properties (Kobayashi *et al.*, 1991). Among them the spiro oxindole is an important structural motif which finds wide applications as antimicrobial and antitumour agents and as inhibitors of the human *NK*1 receptor (Kornet & Thio, 1976). The construction of polyfunctionalized furans, spiro furans and furan fused cycloalkanes are important from the standpoint of synthesis of biologically active natural products such as aflatoxin, asteltoxin, monensin, panacene *etc*., (Schoop *et al.*, 2000).

The bond lengths and angles in the title compound are within normal ranges except those at the spiro junction which reflects the presence of bulky subsituents. The dihedral angle between the five (C5/C6/N1/C7/C8) and six membered (C1–C6) rings in the indole group is 4.15 (8)°. The indole moeity is orthogonal to the furan ring as indicated by the dihedral angle of 83.49 (6)° between them.

The furan ring in the structure adopts a *twisted* conformation with a *psuedo*–twofold axis passing through the C8 atom and C9–C10 bond. The puckering parameters (Cremer & Pople, 1975) for the furan ring are q_2_ = 0.0810 (14)Å, φ_2_ = 310.9 (10)°. The benzene ring is bisectionally oriented to the furan ring, the dihedral angle between them being 52.80 (8)°. The title compound exihibits structural similarities with an already reported related structure (Gayathri *et al.*, 2006.)

The molecular structure is stabilized by intramolecular N2—H2A···O5 bond which generates *S*(6) ring motif. Atom N2 acts as a donor to O1^ii^ generating a centrosymmetric dimer with graph set descriptor of *R*^2^_2_(14) (Bernstein *et al.*, 1995). The bifurcated H bond at O1 facilitates the C13—H13···O1^iii^ bond, which together with a N1—H1(A)···O5^i^ interaction forms a non–centrosymmetric *R*^2^_2_(9) dimer. These non–centrosymmetric dimers aggregate to form *C*^2^_2_[*R*^2^_2_(9)] supramolecular chains running along the *b* axis. The symmetry codes for the interactions are: (i) 1-*x*, -1/2+*y*, 3/2-*z*; (ii) 1-*x*, 1-*y*, 2-*z*; (iii) 1-*x*, 1/2+*y*, 3/2-*z*.

### Experimental

Isatin (1 mmol), aromatic amine (1 mmol), and dimethyl acetylene dicarboxylate (1 mmol) were stirred at room temperature in methanol in the presence of triethylamine (20 mol%) for 4 hrs to give the spirolactones which was filtered out and recrystallized from methanol to afford the pure product (85% yield) as yellow solid.

### Refinement

Positions of the H atoms were localized from the difference electron density maps and their distances were geometrically constrained. The H atoms of amine groups were refined freely with *U*_iso_(H) = 1.2*U*_eq_(N). The atoms bound to the C atoms were treated as riding atoms with d(C—H) = 0.93Å and *U*_iso_(H) = 1.2*U*_eq_(C) for aromatic H; d(C—H) = 0.96Å and *U*_iso_(H) = 1.5*U*_eq_(C) for methyl H. The rotation angle methyl group was optimized by least squares.

### Crystal data

**Table:**

C_19_H_14_N_2_O_5_	*F*(000) = 728
*M**_r_* = 350.32	*D*_x_ = 1.411 Mg m^−^^3^
Monoclinic, *P*2_1_/*c*	Mo *K*α radiation, λ = 0.71073 Å
Hall symbol: -P 2ybc	Cell parameters from 3502 reflections
*a* = 12.1713 (6) Å	θ = 2.4–31.0°
*b* = 13.6144 (7) Å	µ = 0.10 mm^−^^1^
*c* = 10.9602 (6) Å	*T* = 296 K
β = 114.813 (2)°	Block, yellow
*V* = 1648.50 (15) Å^3^	0.30 × 0.25 × 0.20 mm
*Z* = 4	

### Data collection

**Table:**

Bruker SMART APEXII CCD diffractometer	5167 independent reflections
Radiation source: fine–focus sealed tube	3502 reflections with *I* > 2σ(*I*)
Graphite monochromator	*R*_int_ = 0.026
ω– and φ–scans	θ_max_ = 31.0°, θ_min_ = 2.4°
Absorption correction: multi-scan (*SADABS*; Bruker, 2008)	*h* = −17→17
*T*_min_ = 0.969, *T*_max_ = 0.979	*k* = −19→19
20901 measured reflections	*l* = −15→15

### Refinement

**Table:**

Refinement on *F*^2^	Primary atom site location: structure-invariant direct methods
Least-squares matrix: full	Secondary atom site location: difference Fourier map
*R*[*F*^2^ > 2σ(*F*^2^)] = 0.044	Hydrogen site location: inferred from neighbouring sites
*wR*(*F*^2^) = 0.132	H atoms treated by a mixture of independent and constrained refinement
*S* = 1.00	*w* = 1/[σ^2^(*F*_o_^2^) + (0.0665*P*)^2^ + 0.2325*P*] where *P* = (*F*_o_^2^ + 2*F*_c_^2^)/3
5167 reflections	(Δ/σ)_max_ = 0.001
242 parameters	Δρ_max_ = 0.26 e Å^−^^3^
2 restraints	Δρ_min_ = −0.16 e Å^−^^3^

### Special details

**Table:**

Geometry. All s.u.'s (except the s.u. in the dihedral angle between two l.s. planes) are estimated using the full covariance matrix. The cell s.u.'s are taken into account individually in the estimation of s.u.'s in distances, angles and torsion angles; correlations between s.u.'s in cell parameters are only used when they are defined by crystal symmetry. An approximate (isotropic) treatment of cell s.u.'s is used for estimating s.u.'s involving l.s. planes.
Refinement. Refinement of *F*^2^ against ALL reflections. The weighted *R*–factor *wR* and goodness of fit *S* are based on *F*^2^, conventional *R*–factors *R* are based on *F*, with *F* set to zero for negative *F*^2^. The threshold expression of *F*^2^ > σ(*F*^2^) is used only for calculating *R*–factors(gt) *etc*. and is not relevant to the choice of reflections for refinement. *R*–factors based on *F*^2^ are statistically about twice as large as those based on *F*, and *R*–factors based on ALL data will be even larger.

### Fractional atomic coordinates and isotropic or equivalent isotropic displacement parameters (Å^2^)

**Table:**

	*x*	*y*	*z*	*U*_iso_*/*U*_eq_	
C1	0.20517 (13)	0.35795 (13)	0.37822 (16)	0.0565 (4)	
H1	0.2233	0.3054	0.3355	0.068*	
C2	0.13329 (14)	0.43504 (14)	0.30526 (17)	0.0602 (4)	
H2	0.1041	0.4347	0.2121	0.072*	
C3	0.10387 (14)	0.51262 (13)	0.36769 (17)	0.0573 (4)	
H3	0.0555	0.5635	0.3164	0.069*	
C4	0.14643 (12)	0.51452 (11)	0.50668 (16)	0.0494 (3)	
H4	0.1265	0.5660	0.5494	0.059*	
C5	0.21853 (11)	0.43880 (9)	0.57979 (14)	0.0401 (3)	
C6	0.24886 (11)	0.36182 (10)	0.51613 (15)	0.0444 (3)	
C7	0.35751 (11)	0.32910 (9)	0.73933 (15)	0.0440 (3)	
C8	0.28153 (11)	0.42334 (9)	0.72866 (14)	0.0383 (3)	
C9	0.21736 (12)	0.45201 (9)	0.89615 (14)	0.0416 (3)	
C10	0.30798 (11)	0.53001 (8)	0.90518 (13)	0.0355 (3)	
C11	0.35077 (10)	0.50798 (8)	0.81357 (13)	0.0346 (3)	
C12	0.45130 (11)	0.55592 (8)	0.79841 (13)	0.0347 (3)	
C13	0.57815 (14)	0.55042 (11)	0.68384 (18)	0.0541 (4)	
H13A	0.5593	0.6150	0.6455	0.081*	
H13B	0.5951	0.5079	0.6240	0.081*	
H13C	0.6477	0.5537	0.7686	0.081*	
C14	0.26345 (11)	0.64409 (9)	1.05174 (13)	0.0383 (3)	
C15	0.30989 (14)	0.68008 (11)	1.18109 (15)	0.0512 (3)	
H15	0.3924	0.6758	1.2355	0.061*	
C16	0.23301 (17)	0.72265 (13)	1.22943 (18)	0.0649 (5)	
H16	0.2643	0.7472	1.3167	0.078*	
C17	0.11072 (16)	0.72924 (13)	1.1501 (2)	0.0646 (5)	
H17	0.0594	0.7565	1.1844	0.077*	
C18	0.06501 (14)	0.69548 (12)	1.02087 (18)	0.0592 (4)	
H18	−0.0174	0.7007	0.9665	0.071*	
C19	0.14097 (12)	0.65364 (11)	0.97084 (15)	0.0482 (3)	
H19	0.1097	0.6318	0.8823	0.058*	
N1	0.32908 (11)	0.29741 (8)	0.61328 (14)	0.0520 (3)	
H1A	0.3634 (15)	0.2457 (10)	0.5943 (17)	0.062*	
N2	0.34264 (10)	0.60019 (8)	1.00166 (11)	0.0393 (2)	
H2A	0.4110 (10)	0.6321 (10)	1.0165 (15)	0.047*	
O1	0.42614 (9)	0.29188 (7)	0.84405 (12)	0.0569 (3)	
O2	0.19725 (8)	0.39643 (6)	0.78561 (10)	0.0445 (2)	
O3	0.17129 (11)	0.43426 (8)	0.96977 (12)	0.0624 (3)	
O4	0.47595 (8)	0.51218 (7)	0.70423 (10)	0.0448 (2)	
O5	0.50692 (8)	0.62507 (6)	0.86606 (9)	0.0437 (2)	

### Atomic displacement parameters (Å^2^)

**Table:**

	*U*^11^	*U*^22^	*U*^33^	*U*^12^	*U*^13^	*U*^23^
C1	0.0449 (8)	0.0651 (9)	0.0649 (10)	−0.0106 (7)	0.0283 (8)	−0.0261 (8)
C2	0.0438 (8)	0.0854 (12)	0.0511 (8)	−0.0129 (8)	0.0197 (7)	−0.0146 (8)
C3	0.0410 (7)	0.0686 (10)	0.0575 (9)	0.0008 (7)	0.0160 (7)	0.0021 (8)
C4	0.0405 (7)	0.0483 (7)	0.0580 (9)	0.0048 (6)	0.0193 (7)	−0.0054 (6)
C5	0.0299 (6)	0.0394 (6)	0.0516 (7)	−0.0037 (5)	0.0176 (5)	−0.0109 (5)
C6	0.0326 (6)	0.0419 (6)	0.0606 (8)	−0.0062 (5)	0.0214 (6)	−0.0156 (6)
C7	0.0325 (6)	0.0304 (6)	0.0668 (9)	−0.0038 (5)	0.0186 (6)	−0.0062 (6)
C8	0.0319 (6)	0.0315 (5)	0.0542 (8)	−0.0026 (4)	0.0206 (6)	−0.0058 (5)
C9	0.0381 (6)	0.0368 (6)	0.0533 (8)	−0.0040 (5)	0.0224 (6)	0.0004 (5)
C10	0.0325 (5)	0.0308 (5)	0.0442 (7)	−0.0012 (4)	0.0172 (5)	0.0022 (5)
C11	0.0321 (5)	0.0280 (5)	0.0444 (7)	−0.0020 (4)	0.0167 (5)	−0.0012 (5)
C12	0.0330 (5)	0.0303 (5)	0.0428 (6)	−0.0001 (4)	0.0180 (5)	0.0019 (5)
C13	0.0546 (8)	0.0540 (8)	0.0706 (10)	−0.0117 (7)	0.0429 (8)	−0.0084 (7)
C14	0.0401 (6)	0.0340 (6)	0.0454 (7)	−0.0037 (5)	0.0224 (6)	−0.0024 (5)
C15	0.0463 (7)	0.0529 (8)	0.0534 (8)	−0.0050 (6)	0.0199 (7)	−0.0145 (7)
C16	0.0698 (11)	0.0678 (10)	0.0635 (10)	−0.0082 (8)	0.0344 (9)	−0.0273 (8)
C17	0.0623 (10)	0.0629 (10)	0.0865 (12)	−0.0056 (8)	0.0489 (10)	−0.0207 (9)
C18	0.0431 (8)	0.0626 (9)	0.0763 (11)	−0.0004 (7)	0.0293 (8)	−0.0091 (8)
C19	0.0426 (7)	0.0546 (8)	0.0484 (8)	−0.0004 (6)	0.0200 (6)	−0.0041 (6)
N1	0.0444 (6)	0.0364 (6)	0.0737 (8)	0.0024 (5)	0.0234 (6)	−0.0171 (5)
N2	0.0356 (5)	0.0399 (5)	0.0455 (6)	−0.0056 (4)	0.0200 (5)	−0.0062 (4)
O1	0.0459 (5)	0.0375 (5)	0.0743 (7)	0.0017 (4)	0.0126 (5)	−0.0019 (5)
O2	0.0383 (5)	0.0376 (4)	0.0634 (6)	−0.0096 (4)	0.0268 (5)	−0.0070 (4)
O3	0.0679 (7)	0.0621 (7)	0.0739 (7)	−0.0214 (5)	0.0460 (6)	−0.0051 (6)
O4	0.0443 (5)	0.0417 (5)	0.0575 (6)	−0.0095 (4)	0.0304 (5)	−0.0116 (4)
O5	0.0456 (5)	0.0371 (4)	0.0523 (5)	−0.0115 (4)	0.0245 (4)	−0.0072 (4)

### Geometric parameters (Å, º)

**Table:**

C1—C6	1.377 (2)	C11—C12	1.4564 (16)
C1—C2	1.385 (2)	C12—O5	1.2137 (14)
C1—H1	0.9300	C12—O4	1.3304 (15)
C2—C3	1.385 (2)	C13—O4	1.4498 (16)
C2—H2	0.9300	C13—H13A	0.9600
C3—C4	1.388 (2)	C13—H13B	0.9600
C3—H3	0.9300	C13—H13C	0.9600
C4—C5	1.3729 (19)	C14—C15	1.3773 (19)
C4—H4	0.9300	C14—C19	1.3842 (19)
C5—C6	1.3920 (17)	C14—N2	1.4253 (16)
C5—C8	1.4981 (19)	C15—C16	1.381 (2)
C6—N1	1.4083 (19)	C15—H15	0.9300
C7—O1	1.2112 (17)	C16—C17	1.376 (3)
C7—N1	1.3466 (19)	C16—H16	0.9300
C7—C8	1.5571 (17)	C17—C18	1.366 (2)
C8—O2	1.4537 (15)	C17—H17	0.9300
C8—C11	1.4986 (16)	C18—C19	1.380 (2)
C9—O3	1.1847 (16)	C18—H18	0.9300
C9—O2	1.3607 (17)	C19—H19	0.9300
C9—C10	1.5041 (17)	N1—H1A	0.887 (9)
C10—C11	1.3448 (17)	N2—H2A	0.890 (9)
C10—N2	1.3545 (16)		
			
C6—C1—C2	117.66 (14)	O5—C12—O4	124.86 (11)
C6—C1—H1	121.2	O5—C12—C11	123.91 (11)
C2—C1—H1	121.2	O4—C12—C11	111.20 (10)
C3—C2—C1	121.61 (15)	O4—C13—H13A	109.5
C3—C2—H2	119.2	O4—C13—H13B	109.5
C1—C2—H2	119.2	H13A—C13—H13B	109.5
C2—C3—C4	120.11 (16)	O4—C13—H13C	109.5
C2—C3—H3	119.9	H13A—C13—H13C	109.5
C4—C3—H3	119.9	H13B—C13—H13C	109.5
C5—C4—C3	118.64 (14)	C15—C14—C19	119.50 (12)
C5—C4—H4	120.7	C15—C14—N2	119.60 (12)
C3—C4—H4	120.7	C19—C14—N2	120.88 (12)
C4—C5—C6	120.78 (13)	C14—C15—C16	119.51 (14)
C4—C5—C8	130.60 (12)	C14—C15—H15	120.2
C6—C5—C8	108.54 (11)	C16—C15—H15	120.2
C1—C6—C5	121.17 (14)	C17—C16—C15	120.83 (15)
C1—C6—N1	129.20 (13)	C17—C16—H16	119.6
C5—C6—N1	109.60 (12)	C15—C16—H16	119.6
O1—C7—N1	128.04 (12)	C18—C17—C16	119.66 (14)
O1—C7—C8	124.58 (13)	C18—C17—H17	120.2
N1—C7—C8	107.35 (12)	C16—C17—H17	120.2
O2—C8—C11	103.98 (10)	C17—C18—C19	120.11 (15)
O2—C8—C5	111.85 (10)	C17—C18—H18	119.9
C11—C8—C5	117.76 (11)	C19—C18—H18	119.9
O2—C8—C7	105.30 (10)	C18—C19—C14	120.34 (14)
C11—C8—C7	115.18 (10)	C18—C19—H19	119.8
C5—C8—C7	102.35 (10)	C14—C19—H19	119.8
O3—C9—O2	122.23 (12)	C7—N1—C6	111.98 (11)
O3—C9—C10	130.00 (13)	C7—N1—H1A	123.5 (11)
O2—C9—C10	107.70 (11)	C6—N1—H1A	124.2 (11)
C11—C10—N2	130.46 (11)	C10—N2—C14	123.85 (10)
C11—C10—C9	107.25 (11)	C10—N2—H2A	116.6 (10)
N2—C10—C9	121.93 (11)	C14—N2—H2A	117.1 (10)
C10—C11—C12	126.32 (11)	C9—O2—C8	110.27 (9)
C10—C11—C8	110.00 (10)	C12—O4—C13	116.51 (10)
C12—C11—C8	123.66 (11)		
			
C6—C1—C2—C3	−1.3 (2)	C5—C8—C11—C10	122.03 (12)
C1—C2—C3—C4	−0.1 (2)	C7—C8—C11—C10	−117.01 (12)
C2—C3—C4—C5	0.6 (2)	O2—C8—C11—C12	176.23 (10)
C3—C4—C5—C6	0.3 (2)	C5—C8—C11—C12	−59.41 (15)
C3—C4—C5—C8	176.78 (13)	C7—C8—C11—C12	61.55 (16)
C2—C1—C6—C5	2.1 (2)	C10—C11—C12—O5	−2.4 (2)
C2—C1—C6—N1	−175.49 (13)	C8—C11—C12—O5	179.27 (12)
C4—C5—C6—C1	−1.7 (2)	C10—C11—C12—O4	175.70 (12)
C8—C5—C6—C1	−178.88 (12)	C8—C11—C12—O4	−2.62 (16)
C4—C5—C6—N1	176.37 (12)	C19—C14—C15—C16	1.8 (2)
C8—C5—C6—N1	−0.85 (14)	N2—C14—C15—C16	179.97 (14)
C4—C5—C8—O2	73.88 (17)	C14—C15—C16—C17	0.2 (3)
C6—C5—C8—O2	−109.27 (11)	C15—C16—C17—C18	−1.6 (3)
C4—C5—C8—C11	−46.46 (18)	C16—C17—C18—C19	1.0 (3)
C6—C5—C8—C11	130.39 (11)	C17—C18—C19—C14	1.1 (2)
C4—C5—C8—C7	−173.86 (13)	C15—C14—C19—C18	−2.5 (2)
C6—C5—C8—C7	2.99 (12)	N2—C14—C19—C18	179.41 (13)
O1—C7—C8—O2	−65.30 (15)	O1—C7—N1—C6	−177.92 (13)
N1—C7—C8—O2	112.83 (12)	C8—C7—N1—C6	4.03 (15)
O1—C7—C8—C11	48.61 (18)	C1—C6—N1—C7	175.69 (14)
N1—C7—C8—C11	−133.26 (12)	C5—C6—N1—C7	−2.14 (15)
O1—C7—C8—C5	177.64 (12)	C11—C10—N2—C14	151.63 (13)
N1—C7—C8—C5	−4.23 (13)	C9—C10—N2—C14	−36.17 (18)
O3—C9—C10—C11	167.68 (15)	C15—C14—N2—C10	152.11 (13)
O2—C9—C10—C11	−9.33 (14)	C19—C14—N2—C10	−29.79 (18)
O3—C9—C10—N2	−6.1 (2)	O3—C9—O2—C8	−169.38 (13)
O2—C9—C10—N2	176.87 (11)	C10—C9—O2—C8	7.91 (14)
N2—C10—C11—C12	1.5 (2)	C11—C8—O2—C9	−3.79 (13)
C9—C10—C11—C12	−171.60 (11)	C5—C8—O2—C9	−131.88 (11)
N2—C10—C11—C8	179.99 (12)	C7—C8—O2—C9	117.71 (11)
C9—C10—C11—C8	6.91 (14)	O5—C12—O4—C13	0.81 (19)
O2—C8—C11—C10	−2.33 (13)	C11—C12—O4—C13	−177.27 (11)

### Hydrogen-bond geometry (Å, º)

**Table:**

*D*—H···*A*	*D*—H	H···*A*	*D*···*A*	*D*—H···*A*
N1—H1*A*···O5^i^	0.89 (2)	2.19 (2)	3.0268 (16)	157 (2)
N2—H2*A*···O1^ii^	0.89 (1)	2.19 (1)	2.9907 (17)	149 (1)
N2—H2*A*···O5	0.89 (1)	2.39 (2)	2.9691 (16)	123 (1)
C13—H13*A*···O1^iii^	0.96	2.41	3.2999 (18)	153

Symmetry codes: (i) −*x*+1, *y*−1/2, −*z*+3/2; (ii) −*x*+1, −*y*+1, −*z*+2; (iii) −*x*+1, *y*+1/2, −*z*+3/2.
